# Hypoxia-induced let-7f-5p/TARBP2 feedback loop regulates osteosarcoma cell proliferation and invasion by inhibiting the Wnt signaling pathway

**DOI:** 10.18632/aging.103049

**Published:** 2020-04-17

**Authors:** Guangnan Chen, Huijie Gu, Tingting Fang, Kaifeng Zhou, Jun Xu, Xiaofan Yin

**Affiliations:** 1 Department of Orthopaedics, Minhang Hospital, Fudan University, Shanghai 201199, P.R. China; 2 Liver Cancer Institute, Zhongshan Hospital, Fudan University, Shanghai 201100, P.R. China

**Keywords:** osteosarcoma, let-7f-5p, TARBP2, hypoxia, feedback loop

## Abstract

Osteosarcoma (OS) is the most common bone tumor in children and adolescents and is characterized by high metastatic and recurrence rates. In the past, it has been shown that microRNAs may play critical roles in hypoxia-related OS proliferation and invasion. However, the mechanisms by which OS cells acquire this malignant phenotype have remained largely unknown. In the present study, we report that let-7f-5p and TARBP2 were expressed in lower amounts in human OS cell lines when compared with the hFOB normal human osteoblastic cell line; however, both types of cells were repressed by hypoxia. let-7f-5p and TARBP2 significantly inhibited the proliferation and invasion of OS cells. Furthermore, TARBP2 as a downstream and functional target of let-7f-5p regulated the expression of let-7f-5p, and there was a regulatory feedback loop between let-7f-5p and TARBP2. This loop reduced the expression of let-7f-5p and TARBP2 in OS cells to a very low level, which was induced by hypoxia. Furthermore, the hypoxia-induced let-7f-5p/TARBP2 feedback loop contributed to activation of the Wnt signaling pathway. Taken together, our data clearly showed that the feedback loop between let-7f-5p and TARBP2 induced by the hypoxia-promoted OS cell malignant phenotype increased with activation of the Wnt signaling pathway.

## INTRODUCTION

Osteosarcoma (OS) is the most common primary bone cancer and the third most common cancer, occurring predominantly in children and adolescents; it is characterized by rapid growth, and is highly prone to metastasis and recurrence [1, 2]. Patients with non-metastatic OS have a 60%–70% chance of survival 5 years after diagnosis. Although surgical resection and chemotherapy have been shown to significantly improve survival times in OS patients, the outcomes are reduced in OS patients with metastases [2, 3]. Thus, high proliferation and metastasis are the key factors of an OS poor prognosis. Moreover, the molecular mechanism of OS proliferation and metastasis has not yet been fully determined. Additional studies are therefore essential for the development of new prognostic biomarkers and targeted gene therapies.

Hypoxia is a common feature of the cancer microenvironment, and is present in almost all solid tumors, including OS [4]. For solid tumors, hypoxia plays an important role in promoting excessive tumor proliferation and invasion by triggering a series of molecular reactions to favor the formation of a neoplastic microenvironment [5, 6].

MicroRNAs (miRNAs) are a class of non-coding RNAs of approximately 22 nucleotides in length that normally mediate post-transcriptional gene suppression by inhibiting protein translation or cleavage of RNA transcripts in a sequence-specific manner. They play an important role in many biological processes such as development and cell differentiation, apoptosis, proliferation, and senescence [7, 8]. Numerous studies have shown that miRNAs, functioning as either oncogenes or tumor suppressors, have important regulatory functions in biological processes that represent the hallmarks of cancer, such as proliferation, apoptosis, invasion, and metastasis [9].

TARBP2 is a double-stranded RNA binding protein that acts as an RNA-binding subunit on the RNA-induced silencing complex (RISC) involved in miRNA processing and maturation [10, 11]. In recent years, it has been reported that TARBP2 plays an important role in the progression of various tumors [10, 12–14]. Our study found that TARBP2 increases the expression of let-7f-5p in OS cells, suggesting that TARBP2 may promote the maturation of pre-let-7f in these cells. The let-7f-5p miRNA is a member of the let-7 family, which is involved in cell growth, migration, invasion, and angiogenesis in tumors [15–17]. In the present study, we showed that let-7f-5p targeted TARBP2, suggesting there was a regulatory feedback loop between let-7f-5p and TARBP2.

These kinds of regulatory feedback loops, which can further enhance the regulation of genes in tumor behavior, are common in tumors. Similarly, miRNAs frequently form feedback circuits [7, 18], because they are themselves regulated by genes, which they directly or indirectly target. Such self-stabilizing regulatory loop destructions can be central components of epigenetic switches, and serve crucial functions in the process of tumor genesis and metastasis [19, 20]. In the present study, we focused on let-7f-5p and identified a regulatory feedback loop between TARBP2 and let-7f-5p, which could result in proliferation and invasion in OS, depending on the context of cellular hypoxia.

## RESULTS

### let-7f-5p was downregulated in OS cell lines

**Figure 1:**
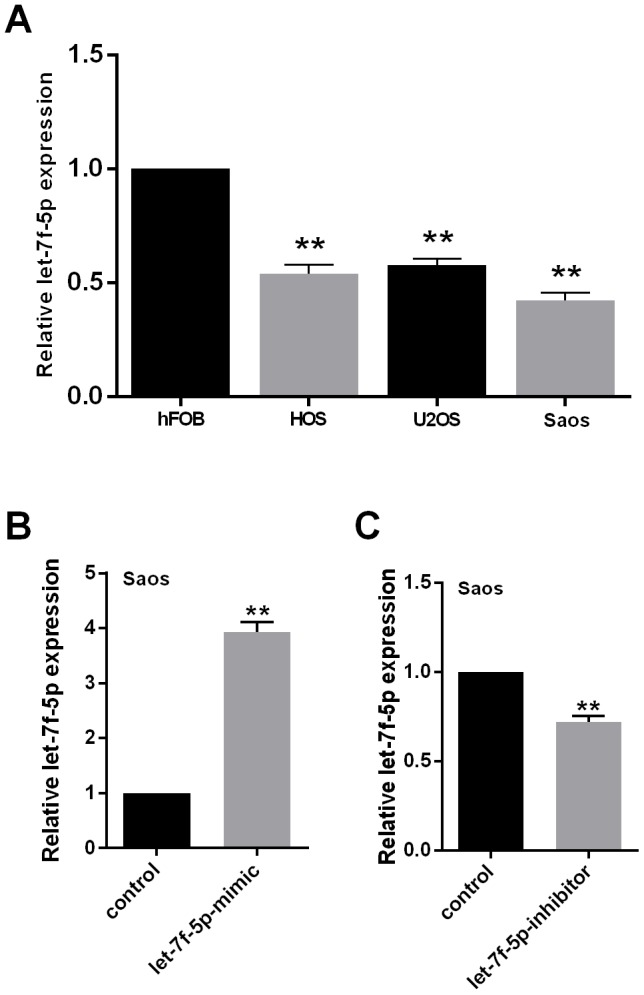
(**A**) let-7f-5p expression was decreased in osteosarcoma (OS) cell lines. qRT-PCR was used to evaluate the endogenous levels of let-7f-5p in hFOB cells and HOS, U2OS, and Saos OS cell lines. (**B**) and (**C**) qRT-PCR was used to quantitate the expression of let-7f-5p following transfection with the let-7f-5p mimic or inhibitor in Saos cells.

To determine the basic characteristics of let-7f-5p, we examined its expression using qRT-PCR in the hFOB normal human osteoblastic cell line and the HOS, U2OS, and Saos human OS cell lines. Compared with hFOB, the expression of let-7f-5p in the OS cell lines was significantly decreased (Figure 1A).

To identify the role played by let-7f-5p in OS development *in vitro*, let-7f-5p expression was downregulated using a let-7f-5p inhibitor in Saos cells, while a let-7f-5p mimic was used for overexpression. *In vivo*, let-7f-5p expression in Saos cells was upregulated by the mimic using a vector of hU6-MCS-CMV-EGFP (let-7f-5p-o) with empty hU6-MCS-CMV-EGFP as a negative control (let-7f-5p-o-control). Knockdown or overexpression of let-7f-5p in Saos cells was successfully detected by qRT-PCR (Figure 1B, 1C).

### let-7f-5p negatively regulated proliferation and invasion of Saos cells *in vitro* and *in vivo*

**Figure 2:**
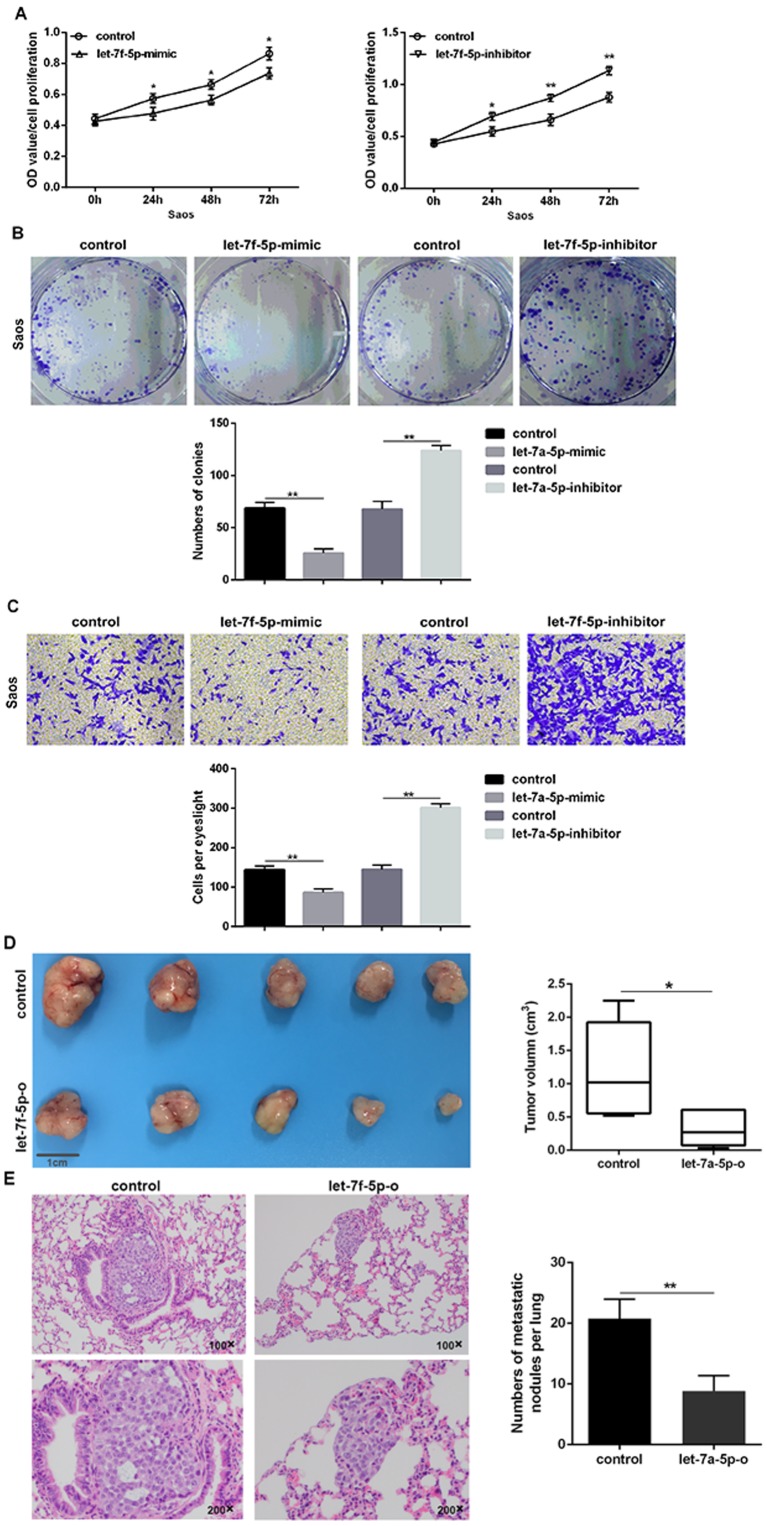
**let-7f-5p suppressed proliferation and invasion of Saos cells *in vitro* and *in vivo*.** (**A**) The CCK8 assay results showed that let-7f-5p suppressed the proliferation of Saos cells *in vitro*. (**B**) The colony formation assay results showed that let-7f-5p suppressed the clonality of Saos cells *in vitro*. (**C**) The Transwell assay results showed that let-7f-5p suppressed the invasion of Saos cells *in vitro*. (**D**) Representative graphs and statistical results of tumor masses harvested 4 weeks after injecting Saos cells subcutaneously into nude mice. (**E**) Representative photographs of lung metastases and statistical results of lung metastatic nodule numbers between the Saos-let-7f-5p-o and Saos-control groups 6 weeks after injecting Saos cells into the lateral tail veins of nude mice (magnification: 100× and 200×).

Saos cells were treated with or without the mimic or inhibitor of let-7f-5p. Upregulation of let-7f-5p in Saos cells resulted in a significant decrease in cell proliferation, clonality, and invasion when compared with its negative counterpart, while downregulation of let-7f-5p in Saos cells promoted increased cell proliferation, clonality, and invasion when compared to its control *in vitro* (Figure 2A–2C).

Saos cells stably overexpressing let-7f-5p (Saos-let-7f-5p-o) and corresponding control Saos cells (Saos-let-7f-5p-o-control) were injected subcutaneously into nude mice to establish a subcutaneous tumor model. Subcutaneous tumors of Saos cells overexpressing let-7f-5p were significantly smaller than the control group (Figure 2D). To determine the correlation between let-7f-5p and the ability of OS cells to circulate as a seed transfer, we established a model of metastatic OS. We injected Saos cells stably expressing let-7f-5p and control cells into the lateral tail veins of nude mice and determined possible evidence of circulating seeding metastasis. The results showed that lung metastases occurred in Saos cells in the control group, while lung metastases were decreased in the Saos-let-7f-5p-o group (Figure 2E). Taken together, these results suggested that let-7f-5p inhibited the proliferation and invasion of OS cells *in vitro* and *in vivo*.

### Hypoxia inhibited let-7f-5p expression in OS cell lines and and promotes the targeted inhibition of TARBP2 by let-7f-5p

**Figure 3:**
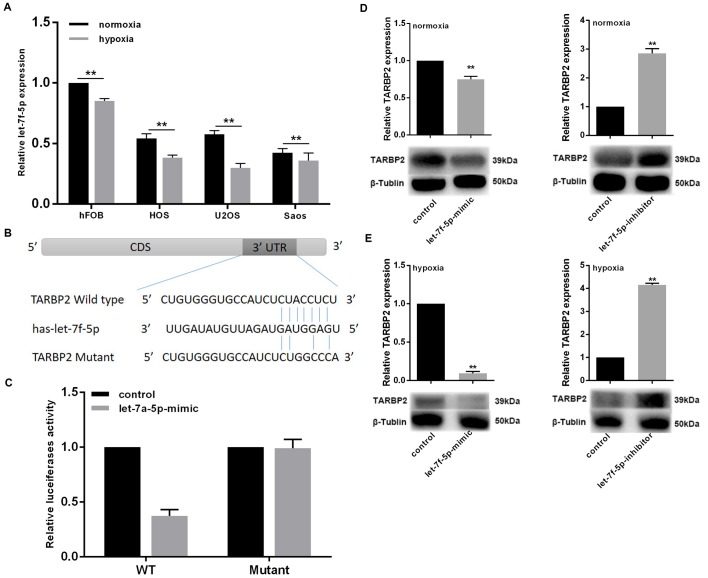
**Hypoxia inhibited let-7f-5p expression in OS cell lines and promotes the targeted inhibition of TARBP2 by let-7f-5p.** (**A**) let-7f-5p downregulated endogenous TARBP2 mRNA and protein expression in Saos cells. (**B**) Sequence alignment of the predicted interactions of let-7f-5p and its predictive target sites within the 3′-UTR of TARBP2. (**C**) The let-7f-5p mimics reduced luciferase activities controlled by the wild type 3′-UTR of TARBP2 but did not affect luciferase activity controlled by the mutant 3′-UTR of TARBP2. (**D**) let-7f-5p overexpression or knockdown reduced or increased the expression of endogenous TARBP2, respectively. (**E**) The regulation of let-7f-5p on TARBP2 expression is more pronounced in hypoxia condition.

To verify the effect of hypoxia on let-7f-5p expression in OS cells, OS cell lines were exposed to 2% O_2_ with 5% CO_2_ at 37°C for 48 h. let-7f-5p expression was decreased in OS cell lines (Figure 3A). The function of miRNAs mainly involves mediating post-transcriptional targeted gene suppression. To identify the molecular mechanisms by which let-7f-5p regulates OS cell proliferation and invasion, we used the TargetScan database (http://www.targetscan.org) to predict binding sites for let-7f-5p within TARBP2. TARBP2 is a double-stranded RNA-binding protein that acts as an RNA-binding subunit on RISC [21], where it mediates the biological functions of miRNAs [10, 11]. In recent years, it has been reported that TARBP2 plays an important role in the progression of many tumors [10, 12, 22]. We targeted the let-7f-5p binding site of the TARBP2 3′-UTR (Figure 3B) and constructed plasmids containing wild-type and mutant TARBP2 to transfect Saos cells. The luciferase reporter assays showed that luciferase activity was repressed in Saos-let-7f-5p-mimic cells, whereas the let-7f-5p-mutant plasmids did not affect reporter gene activity, indicating that let-7f-5p bound to the 3'-UTR of TARBP2 mRNA, and TARBP2 was a downstream target of let-7f-5p (Figure 3C). Furthermore, we detected the expression of TARBP2 after let-7f-5p expression changes in Saos cells in normoxia or hypoxia condition. The results showed that let-7f-5p overexpression or knockdown reduced or increased the expression of endogenous TARBP2, respectively, suggesting that complementary binding of let-7f-5p to the 3'-UTR of TARBP2 mRNA resulted in degradation of the *TARBP2* target gene. In addition, the regulation of let-7f-5p on TARBP2 expression is more pronounced in hypoxia condition (Figure 3D, 3E). Taken together, these results suggested that hypoxia decreases let-7f-5p expression and promotes the targeted inhibition of TARBP2 by let-7f-5p.

### Hypoxia inhibited TARBP2 expression in OS cell lines and let-7f-5p/TARBP2 comprises a feedback loop induced by hypoxia

**Figure 4:**
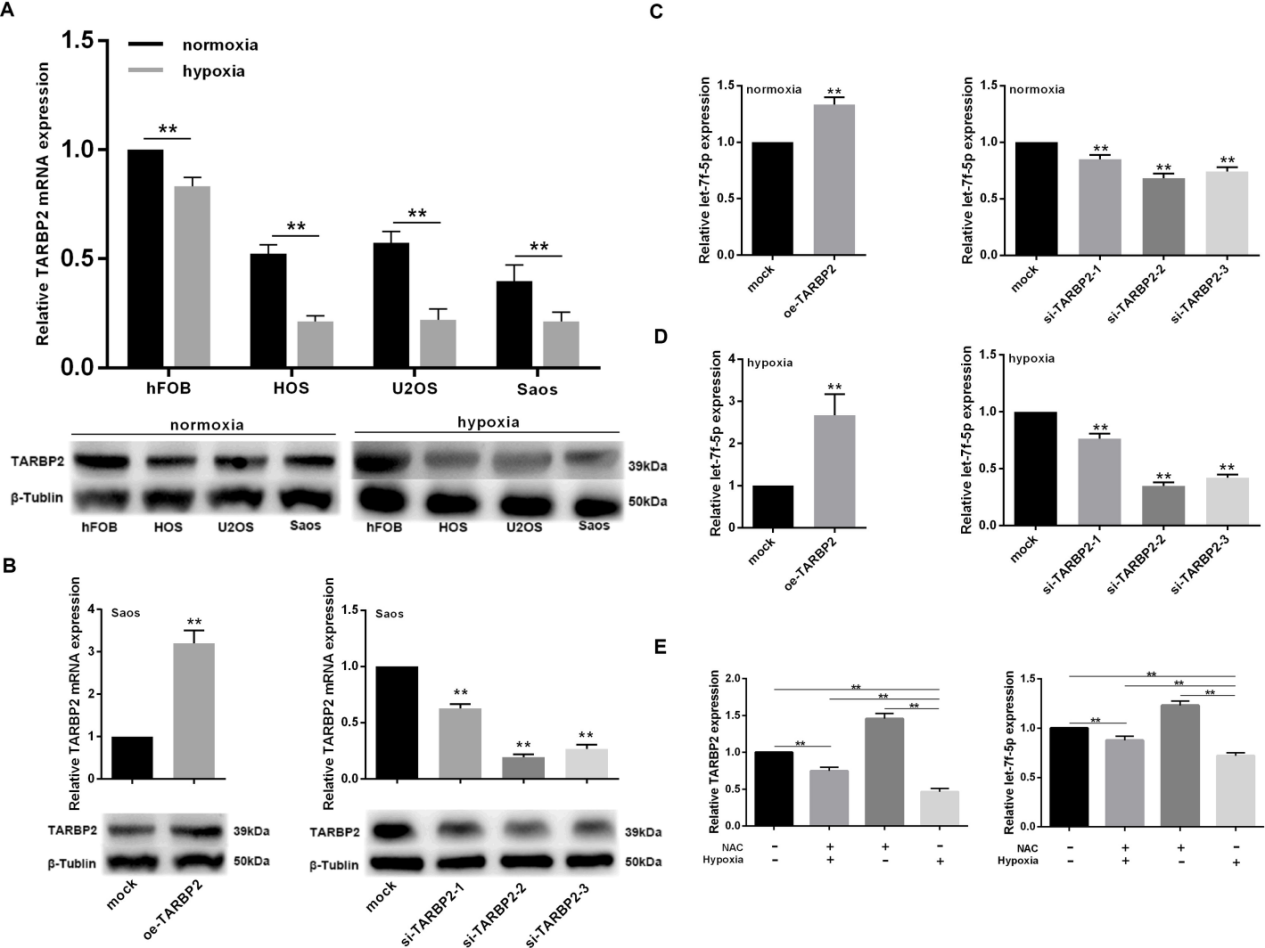
**let-7f-5p/TARBP2 comprises a feedback loop induced by hypoxia.** (**A**) TARBP2 expression was decreased in OS cell lines in a hypoxic environment and TARBP2 was downregulated in OS cell lines. qRT-PCR and western blotting were used to quantitate the endogenous levels of TARBP2 in hFOB cells and HOS, U2OS, and Saos OS cells. (**B**) qRT-PCR and western blotting were used to evaluate the expression of TARBP2 following transfection with the TARBP2 overexpression plasmid or siRNA in Saos cells. (**C**) Upregulation of TARBP2 in Saos cells showed a significant decrease in let-7f-5p expression when compared with its negative counterpart, while downregulation of TARBP2 in Saos cells showed increased let-7f-5p expression when compared with control cells. (**D**) The regulation of TARBP2 on let-7f-5p expression is more pronounced in hypoxia condition. (**E**) Hypoxia inhibited let-7f-5p expression is caused by reactive oxygen species (ROS) accumulation.

To confirm the effect of hypoxia on TARBP2 expression in OS cells, the cell lines were exposed to 2% O_2_ with 5% CO_2_ at 37°C for 48 h. The results showed that TARBP2 expression decreased in OS cell lines. To determine the basic characteristics of let-7f-5p, we examined its mRNA and protein expression in the hFOB normal human osteoblastic cell line and in HOS, U2OS, and Saos human OS cell lines. Compared with hFOB, the expression of TARBP2 in the OS cell lines was significantly decreased (Figure 4A).

To investigate the role played by TARBP2 in OS development *in vitro*, TARBP2 expression was downregulated using TARBP2 siRNA in Saos cells, while the TARBP2 overexpression plasmid was used for overexpression. Knockdown or overexpression of TARBP2 in Saos cells was successfully detected by qRT-PCR and western blotting (Figure 4B).

Furthermore, we detected the expression of let-7f-5p after TARBP2 expression changes in Saos cells in normoxia or hypoxia condition. The results showed that upregulation of TARBP2 in Saos cells resulted in a significant increase in let-7f-5p expression, when compared with its negative counterpart, while downregulation of TARBP2 in Saos cells showed decreased let-7f-5p expression, when compared with control cells, suggesting that TARBP2 could promote the expression of let-7f-5p (Figure 4C). In addition, the regulation of TARBP2 on let-7f-5p expression is more pronounced in hypoxia condition (Figure 4D). We use NAC (N-acetyl-L-cysteine), which is a ROS inhibitor to test the potential effect of ROS on feedback loop between let-7f-5p and TARBP2. The result showed that hypoxia inhibited let-7f-5p expression is caused by reactive oxygen species (ROS) accumulation (Figure 4E). Taken together, these results suggested that hypoxia decreases TARBP2 expression and promotes the regulation of let-7f-5p by TARBP2, and there was a regulatory feedback loop between let-7f-5p and TARBP2. This loop reduced the expression of let-7f-5p and TARBP2 in OS cells to a very low level induced by hypoxia, which was caused by reactive oxygen species (ROS) accumulation.

### let-7f-5p/TARBP2 feedback loop involved in the proliferation and invasion of OS cells

**Figure 5:**
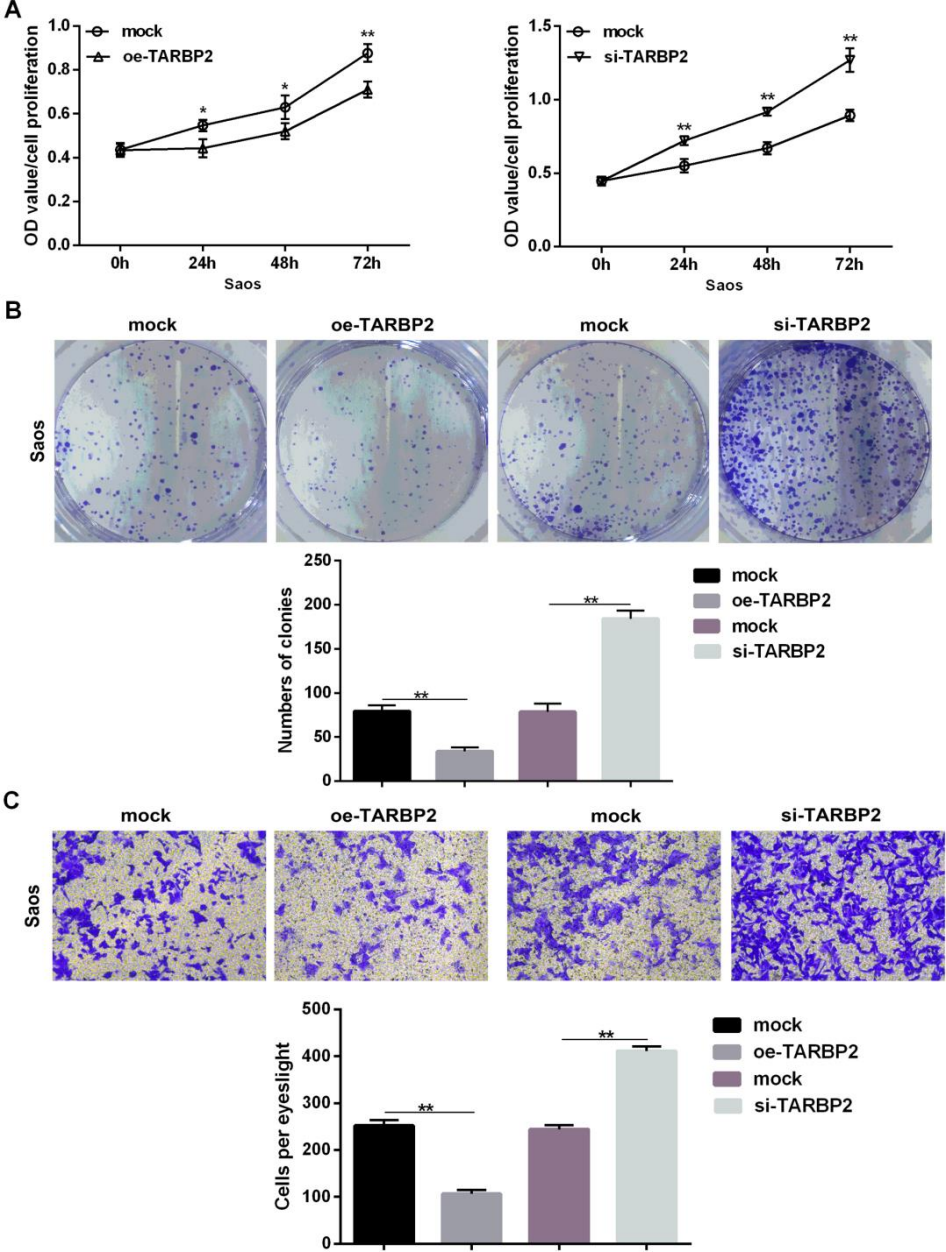
**TARBP2 suppressed proliferation and invasion of Saos cells *in vitro*.** (**A**) The CCK8 assay showed that TARBP2 suppressed proliferation of Saos cells *in vitro*. (**B**) The colony formation assay showed that TARBP2 suppressed the clonality of Saos cells *in vitro*. (**C**) The Transwell assay showed that TARBP2 suppressed the invasion of Saos cells *in vitro*.

Upregulation of TARBP2 in Saos cells showed a significant decrease in cell proliferation, clonality, and invasion when compared with its negative counterpart, while TARBP2 downregulated in Saos cells promoted increased cell proliferation, clonality, and invasion when compared with control cells *in vitro* (Figure 5A–5C). Furthermore, we determined the expression levels of let-7f-5p following TARBP2 expression changes in Saos cells. TARBP2 overexpression or knockdown increased or reduced the expression of endogenous let-7f-5p, respectively, suggesting that TARBP2 was a downstream and functional target of let-7f-5p, and could regulate the expression of let-7f-5p, indicating the presence of a regulatory feedback loop between let-7f-5p and TARBP2 (Figure 5D). Because TARBP2 is an RNA-binding subunit on RISC involved in miRNA processing and maturation [10, 11], we speculated that TARBP2 promotes the maturation of pre-let-7f to increase the expression of let-7f-5p in OS cells. Taken together, our results showed that the let-7f-5p/TARBP2 feedback loop was necessary for the regulation and biological effects of proliferation and invasion of OS cells.

### The hypoxia-induced let-7f-5p/TARBP2 feedback loop inhibited the Wnt signaling pathway in OS cells

**Figure 6:**
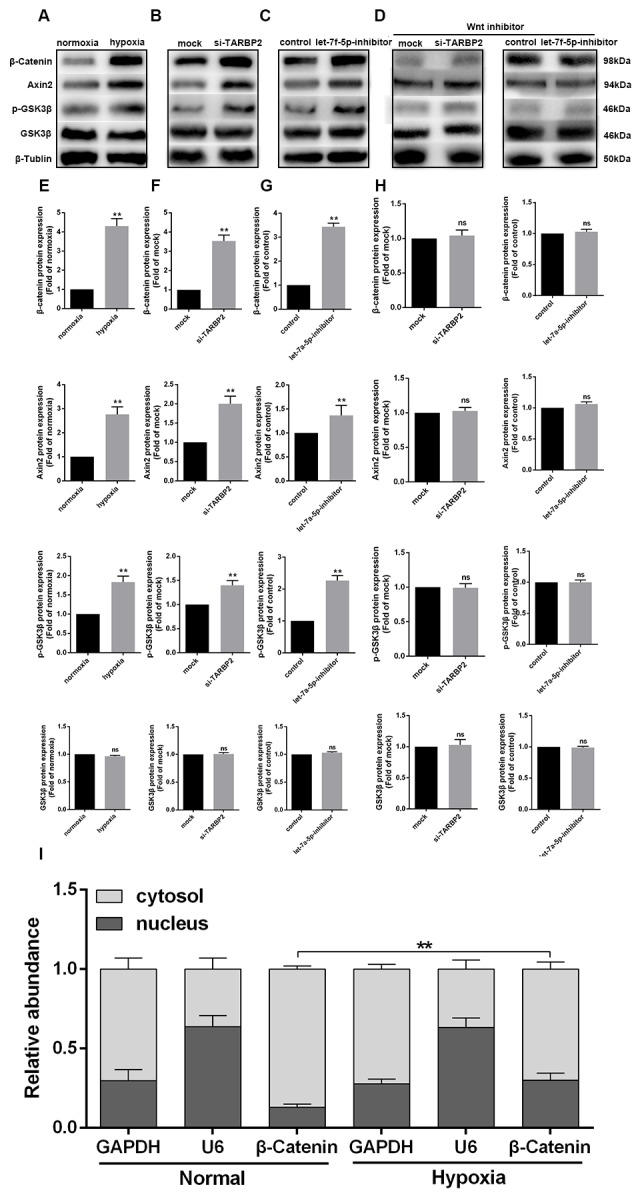
**The hypoxia-induced let-7f-5p/TARBP2 feedback loop inhibited the Wnt signaling pathway in OS cells.** (**A**, **E**) Western blot analyses of the protein expression levels of β-catenin, Axin2, p-GSK3β, and GSK3β in Saos cells under normoxia or hypoxia conditions. (**B**, **F**) Western blot analyses of the protein expression levels of β-catenin, Axin2, p-GSK3β, and GSK3β in Saos cells following transfection with TARBP2 siRNA or control oligonucleotide. (**C**, **G**) Western blot analyses of the protein expression levels of β-catenin, Axin2, p-GSK3β, and GSK3β in Saos cells following transfection with the let-7f-5p inhibitor or control oligonucleotide. (**D**, **H**) Wnt inhibitor could abolish TARBP2 siRNA and let-7f-5p inhibitor-induced Wnt signal pathway activation. (**I**) β-catenin is mainly located in the cytoplasm, and hypoxia promoted the transfer of β-catenin to the nucleus.

**Figure 7:**
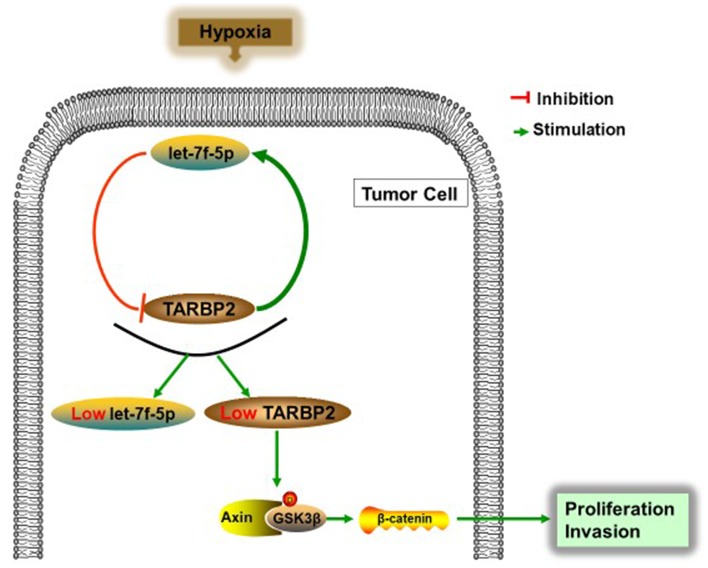
**Schematic representation of the let-7f-5p/TARBP2 regulatory feedback loop in OS cells, which is induced by hypoxia and reduces the expression of let-7f-5p and TARBP2 in OS cells.** A hypoxia-induced let-7f-5p/TARBP2 feedback loop regulates OS cell proliferation and invasion by inhibiting the Wnt signaling pathway.

Hypoxia is common in malignant solid tumors and often contributes to the generation of malignant tumor cells because it activates many downstream signaling cascades, including the Wnt signaling pathway, that result in the growth of human tumors, with the cell cycle, invasion, and apoptosis playing key roles [23–26]. However, the specific biological mechanisms between hypoxia and the Wnt signaling pathway remain unknown. In the present study, we characterized the protein expression levels of β-catenin, Axin2, p-GSK3β, and GSK3β in Saos cells under conditions of normoxia or hypoxia. Figure 6A shows that the expression levels of β-catenin, Axin2, and p-GSK3β were increased in Saos cells grown in hypoxia conditions. TARBP2 siRNA and let-7f-5p inhibitor also remarkably increased protein expression of β-catenin, Axin2, and p-GSK3β (Figure 6B, 6C). In addition, Wnt inhibitor could abolish TARBP2 siRNA and let-7f-5p inhibitor-induced Wnt signal pathway activation (Figure 6D). Furthermore, we detected separated cytoplasm and nuclear RNA by qRT-PCR and found that β-catenin is mainly located in the cytoplasm, and hypoxia promoted the transfer of β-catenin to the nucleus (Figure 6I). These results strongly suggested that let-7f-5p and TARBP2 specifically inhibited hypoxia-induced activity of the Wnt signaling pathway. Taken together, we concluded that hypoxia activated the Wnt pathway by inducing the let-7f-5p/TARBP2 feedback loop in Saos cells (Figure 7).

## DISCUSSION

OS is one of the most common solid tumors of the bone characterized by occult onset, rapid growth, high metastasis, and recurrence in children and adolescents [1]. Although chemotherapy to supplement surgical resection has been shown to significantly improve survival times in patients with OS, the chance of survival for 5 years is still no more than 60%–70% due to tumor rapid growth, invasiveness, and metastasis [2, 3]. Thus, high metastasis and recurrence are the key factors contributing to a poor prognosis for OS. Moreover, the molecular mechanism of OS growth, metastasis, and recurrence has not yet been fully elucidated. Identifying biologically important molecular markers and pathways to provide the basis for new therapeutic approaches is therefore essential for the treatment of patients with OS.

miRNAs are small molecules consisting of 17–22 nucleotides that play important roles in a variety of biological processes, including development, differentiation, cell apoptosis, proliferation, invasion, and cellular senescence [7–9]. They function in the transcriptional and post-transcriptional regulation of gene expression by binding to the 3′-UTR of target mRNAs, which suppresses protein translation [27]. Previous studies have reported that miRNAs are associated with multiple normal biological processes, while aberrant miRNA expression has been associated with oncogenesis and tumor suppression [28]. In the present study, we focused on let-7f-5p, which is located at 9q22.3 and is a member of let-7 family. let-7f has been shown to play an important role in cell growth, migration, invasion, and angiogenesis in tumors [15, 16]. However, the molecular mechanism by which let-7f-5p regulates OS cell proliferation and invasion remains largely unknown. Here, we provide evidence that let-7f-5p expression is significantly downregulated in OS cells, and that let-7f-5p acts to promote the proliferation and invasion of OS cells, further supporting the concept that let-7f-5p inhibits tumor development. Moreover, we identified TARBP2 as a downstream and functional target of let-7f-5p in OS cells.

Accumulating evidence has shown that the expression levels of TARBP2 are frequently deregulated in various human tumors, such as breast cancer, lung cancer, melanoma, and ewing sarcoma [10, 12–14, 22]. Moreover, many studies have further reported that TARBP2 plays an oncogenic or tumor suppressor role in many tumors, both *in vitro* and *in vivo* [10, 12, 13, 22]. Decreased expression of TARBP2 results in miRNA decreases in human cancers [12, 29–31]. However, further studies are required to investigate the underlying molecular mechanisms of TARBP2 in human cancers. In the current study, we found that highly expressed TARBP2 decreased the proliferative and invasive abilities of OS cells. We also showed that let-7f-5p targeted TARBP2, suggesting the existence of a regulatory feedback loop between let-7f-5p and TARBP2. This loop reduced the expression of let-7f-5p and TARBP2 in tumor cells to a low level, which hindered the tumor suppressive effects of let-7f-5p and TARBP2. Furthermore, this process was induced by the hypoxic environment of the tumor.

Hypoxia is a fundamental microenvironmental component of solid tumors, which is associated with resistance to therapy, poor survival, and a malignant phenotype [32]. However, the hypoxia-induced downstream signaling pathways that regulate the proliferation and invasion of OS cells are largely unknown. In the present study, we showed the hypoxic regulation of the Wnt signaling pathway in a context-dependent manner in OS cells, and the role of this process in the maintenance of an aggressive tumor phenotype. Furthermore, regulation of the Wnt signaling pathway by inhibiting the feedback loop between let-7f-5p and TARBP2 induced by hypoxia, which was caused by reactive oxygen species (ROS) accumulation. However, further studies are needed to fully delineate the roles of these components in OS malignancy.

## MATERIALS AND METHODS

### Cell culture and reagents

Four human cell lines were used in this study. The hFOB normal human osteoblastic cell line and the Saos, HOS, and U2OS human OS cell lines were purchased from the Shanghai Institute of Cell Biology, Chinese Academy of Sciences (Shanghai, China). The cells were cultured in Dulbecco’s Modified Essential Medium (DMEM) or RPMI 1640 with high glucose (Gibco BRL, Gaithersburg, MD, USA) supplemented with 10% fetal bovine serum (FBS; Gibco BRL), 100 U/mL penicillin, and 100 mg/mL streptomycin. The cells were incubated at 37°C in a humidified atmosphere containing 5% CO_2_. For hypoxic conditions, the cells were exposed to 2% O_2_ with 5% CO_2_ at 37°C. After incubation for the desired periods, the cells were harvested for subsequent experiments.

### Quantitative real-time polymerase chain reaction (qRT-PCR)

TRIzol^®^ reagent (Invitrogen, Carlsbad, CA, USA) was used to isolate total RNA, cytoplasm RNA, nuclear RNA or miRNA from cell lines according to the manufacturer’s instructions. For miRNA, qRT-PCR was performed in triplicate by the SYBR Green PCR method using an All-in-One miRNA qPCR Detection kit (GeneCopoeia, Rockville, MD, USA). For mRNA, RNA was reverse-transcribed using the PrimeScript RT Master Mix (Takara, Shiga, Japan) and SYBR Green fluorescence-based qRT-PCR was performed according to the manufacturer’s instructions (Takara). The threshold cycle values were determined using the 2^-ΔΔCt^ cycle threshold method after normalization to the internal control (U6 or glyceraldehyde 3-phosphate dehydrogenase). The primers were all synthesized and purchased from Shenggong Company (Shanghai, China). The specific primers were as follows: let-7f-5p: 5′-CGC GCG TGA GGT AGT AGA TTG T-3′ (forward) and 5′-AGT GCA GGG TCC GAG GTA TT-3′ (reverse), U6: 5′-CAA ATT CGT GAA GCG TTC CAT AT-3′(forward) and 5′-GTG CAG GGT CCG AGG T-3′ (reverse), TARBP2: 5′-TTG AGG AGC TGA GCC TGA GTG G-3′ (forward) and 5′-TTG CTG CCT GCC ATG ATC TTG AG-3′ (reverse), GAPDH: 5′-TGA CTT CAA CAG CGA CAC CCA-3′ (forward) and 5- CAC CCT GTT GCT GTA GCC AAA -3′ (reverse).

### Western blotting

Western blotting was performed as previously described [33]. Blocked membranes with proteins were incubated with primary antibody overnight at 4°C, washed three times with TBST, and incubated with secondary antibody for 2 h at room temperature. The protein bands were detected with an enhanced chemiluminescence kit (Cell Signaling Technologies, Danvers, MA, USA). The antibodies used were anti-TARBP2 (1:1,000), anti-β-catenin (1:1,000), anti-Axin2 (1:1,000), anti-phospho-GSK3β (p-GSK3β) (1:1,000), anti-GSK3β (1:1,000), and anti-β-tubulin (1:2,000) (all from Abcam, Cambridge, MA, USA).

### Cell transfection

Oligonucleotides including the miRIDIAN let-7f-5p hairpin inhibitor, mimic, and negative control (Thermo Fisher Scientific, Cleveland, OH, USA) for inhibition and restoration of let-7f-5p were used in this study. hU6-MCS-CMV-EGFP was used as a vector for ectopic overexpression of let-7f-5p (let-7f-5p-o) and the negative control (let-7f-5p-o-control). The small interfering RNA (siRNA) targeting human TARBP2 (si-TARBP2) and TARBP2 ectopic expression (oe-TARBP2) as well as their negative (mock) controls were designed and synthesized by GeneChem (Shanghai, China). All constructs and oligonucleotides were transfected into Saos cells using Lipofectamine 2000 (Invitrogen), according to the manufacturer’s instructions. Transfection efficiencies were evaluated by performing qRT-PCR and western blotting at 48 h post-transfection. The siRNA sequences were as follows: siRNA1, UACUGAAUUUCGUAGAGAAUCCCAGGU; siRNA2, GCAGCAUAUUUAUUCCAGGCUCUAGUA; siRNA3, GCUGAGGCUCUGUGGUCCUUGGCUCCU [13].

### Cell proliferation assays

A total of 4,000 cells/well were seeded in 96-well culture plates in triplicate. At the indicated time points, 10 μL of CCK-8 solution (Dojindo Molecular, Tokyo, Japan) was added and after incubation for another 2 h, the OD values at 450 nm were measured using an Infinite 200 (Tecan Life Sciences, Manndorf, Switzerland). Three independent experiments were performed.

### Invasion assay

Cell invasion assays were performed in 24-well Transwell chambers with an 8.0 μm pore polycarbonate membrane insert (Corning, Corning, NY, USA) as previously described [33]. Briefly, 1 × 10^5^ cells in serum-free medium were placed into the upper chamber insert with Matrigel (BD Biosciences, San Jose, CA, USA) for 48 h at 37°C. The lower chamber was filled with 600 μL DMEM or RPMI 1640 with high glucose (Gibco BRL) supplemented with 10% FBS (Gibco BRL). The cells on the undersurface were stained with 0.1% Crystal Violet for 15 min at room temperature, and counted (10 fields) using a 100× objective.

### Colony formation assay

OS cells were equally seeded (2,000 cells/well) in a 6-well plate. Culture medium was replaced every 3 days. After incubation for 11 days, the colonies were stained with 0.1% Crystal Violet for 15 min at room temperature, washed, and photographed.

### Animal assay

All animal experiments were performed in accordance with current guidelines and followed a protocol approved by the Animal Care and Use Committee of Fudan University, China. The nude mice subcutaneous tumor model and metastatic model were established as previously described [33]. Athymic nude BALB/c mice (4 weeks of age, Shanghai Laboratory Animal Center, Chinese Academy of Science, Shanghai, China) were raised under specific pathogen-free conditions. Briefly, the mice were inoculated subcutaneously with 1 × 10^7^ single cell suspensions of Saos cells in 0.1 mL phosphate-buffered saline and injected subcutaneously into the flanks of the right anterior limbs or injected into the lateral tail veins of 4-week-old BALB/c nude mice (n = 5 per group). Subcutaneous tumors were harvested 4 weeks after injection, and each tumor volume was calculated. The metastatic model mice were sacrificed at 6 weeks and the lung metastases were evaluated.

### Luciferase reporter assay

The Dual Luciferase Reporter assay was performed as previously described with the following modifications [33]. Briefly, wild-type and mutant 3′-UTRs of TARBP2 were amplified and cloned downstream of the luciferase gene within the pGL3/luciferase vector (Promega, Madison, WI, USA), with the Renilla luciferase plasmid (pRL-TK, Promega) co-transfected as a control. At 36 h after transfection, the luciferase activities of the transformed cells were measured, using a Dual Luciferase Reporter assay kit (Promega) at room temperature, according to the manufacturer's instructions.

### Statistical analyses

Statistical analyses were performed using SPSS statistical software for Windows, version 19.0 (SPSS, Chicago, IL, USA) and GraphPad Prism 6.0 (GraphPad, La Jolla, CA, USA). Student’s *t*-tests, Pearson’s χ2 tests, or Fisher’s exact tests were used to determine significant differences between the study groups. A two-sided value of p < 0.05 was considered statistically significant. All *in vitro* experiments were performed in triplicate and repeated at least three times.
